# An Overview of Vaginoplasty Techniques: Spotlight on the Efficacy of the Horseshoe Labia Minora Flap

**DOI:** 10.1055/s-0045-1809412

**Published:** 2025-06-12

**Authors:** Rasheedha Begum, Manimegala Philip, Mahadevan Kandasamy, Sridevi Shanmugam, Sugumar Muthachari

**Affiliations:** 1 Department of Plastic, Reconstructive and Faciomaxillary Surgery, Madras Medical College, Chennai, Tamil Nadu, India; 2 Department of Plastic and Reconstructive Surgery, Government Kilpauk Medical College, Kilpauk, Chennai, Tamil Nadu, India; 3 Institute of Research and Rehabilitation of Hand and Department of Plastic Surgery, Stanley Medical College, Chennai, Tamil Nadu, India

**Keywords:** vaginoplasty, Mayer–Rokitansky–Küster–Hauser syndrome, McIndoe procedure, pudendal flap, Singapore flap, labia minora flap

## Abstract

**Background:**

Mayer–Rokitansky–Küster–Hauser (MRKH) syndrome involves vaginal agenesis and variable uterine development, often accompanied by renal, skeletal, and auditory anomalies. The primary objective of treatment is to reconstruct a neovagina that closely resembles the natural anatomy, enabling normal sexual function. This study evaluates various vaginoplasty techniques performed at our institution and their outcomes.

**Materials and Methods:**

This retrospective study, conducted from January 2012 to January 2024, includes patients who underwent different types of vaginoplasty, including the McIndoe, Singapore flap, islanded pudendal flap, labia minora flap, and horseshoe modification of the labia minora flap. Procedures were chosen based on clinical examination. Outcomes were assessed using an institutional scoring system evaluating vaginal length, introitus diameter, neovaginal skin quality, and pain during intercourse.

**Results:**

Twenty patients of primary vaginal agenesis (aged 18–27) diagnosed clinically and confirmed with pelvic MRI underwent vaginoplasty by the same surgical team. One (14.28%) patient of the McIndoe procedure experienced graft contracture, which was released and regrafted, while another (14.28%) had partial graft loss managed conservatively. No significant complications occurred in the remaining patients. All used a postoperative vaginal mould for three months. The average follow-up period was 18 months, with neovaginal length ranging from 6 to 10 cm.

**Conclusion:**

The horseshoe modification of the labia minora flap offers a reliable, effective approach for vaginal reconstruction in cases of MRKH syndrome. This technique fulfils the basic tenets of plastic surgery, namely the restoration of form and function. The simplicity of the procedure and relatively short learning curve render it an attractive flap option even for the novice plastic surgeon.

## Introduction

Mayer–Rokitansky–Küster–Hauser (MRKH) syndrome is characterized by congenital vaginal absence and variable uterine development. It affects approximately 1 in 4,000 to 5,000 female births and may include renal, skeletal, or auditory abnormalities. Patients typically have a normal female phenotype, 46, XX karyotype, normal ovaries, and normal secondary sexual characteristics. The first clinical sign is primary amenorrhea. Secondary sexual characteristics such as thelarche and pubarche are normal, and they also have normal external genitalia. 1 2 In some cases, there may be a vaginal dimple instead of a fully developed vagina. The primary treatment goal is neovaginal construction that mimics natural anatomy to support sexual activity without discomfort. The factors required for a reconstructed neovagina include good sensation, lubrication, elasticity, adequate length, width, angle of inclination, and minimal scarring. Additionally, the neovagina should require minimal or no dilatation and should not predispose to stenosis or contracture.

A wide array of techniques for vaginal reconstruction have been described in the literature; however, no single approach has emerged as the definitive standard. Each technique presents its own set of benefits and drawbacks, and selection is often guided by individual patient anatomy, surgical goals, and the presence of complicating factors such as prior radiation or scarring. Fasciocutaneous flaps, which were described earlier, include:

- Perineal artery axial flap of Hagerty. 3
- The Malaga flap by Giraldo. 4 5 6 7
- The Singapore flap of Wee and Joseph. 8
- Modified Singapore flap of Karl Podratz and Woods. 9
- Lotus petal flaps in vulvovaginal reconstruction of Niranjan raised from either the hairy skin of the labia majora or lateral to it. 10

This study evaluates the outcomes of different vaginoplasty techniques performed at our institution.

## Materials and Methods

**Fig. 1:**
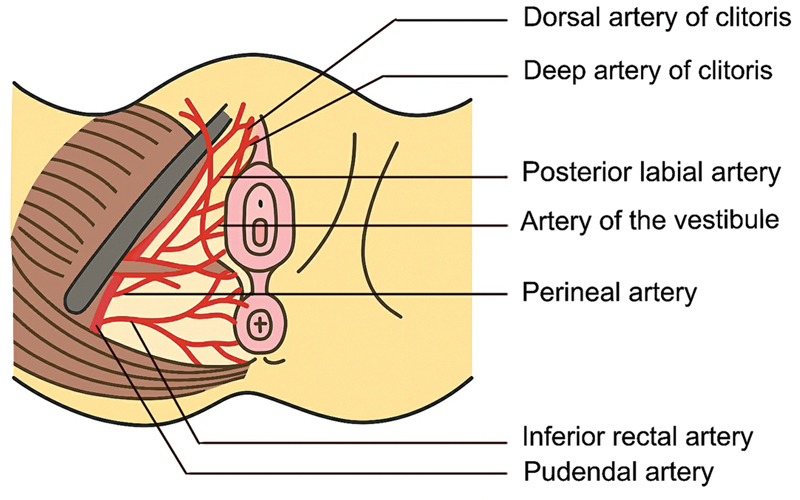
Blood supply of the perineum.

This retrospective analysis includes procedures conducted from January 2012 to January 2024. Technique selection was based on clinical findings and patient preferences. Patients with well-developed labia minora and those who did not want a conspicuous scar in the thigh were offered the option of horseshoe modification of labia minora flap. Horseshoe labia minora flap was preferred for patients with well-developed labia minora and those avoiding thigh scars. The McIndoe procedure was used when the labia minora were underdeveloped or absent. Other options included the Singapore flap and islanded pudendal flap. Fig. 1 shows the blood supply to the perineum. Written informed consent was obtained from all patients prior to the surgical intervention, as well as for the use of clinical materials such as photographs and videos for research and publication. All patients underwent counselling by a psychologist before and after the procedure, as all patients with MRKH syndrome are known to be psychologically distressed.

**Fig. 2:**
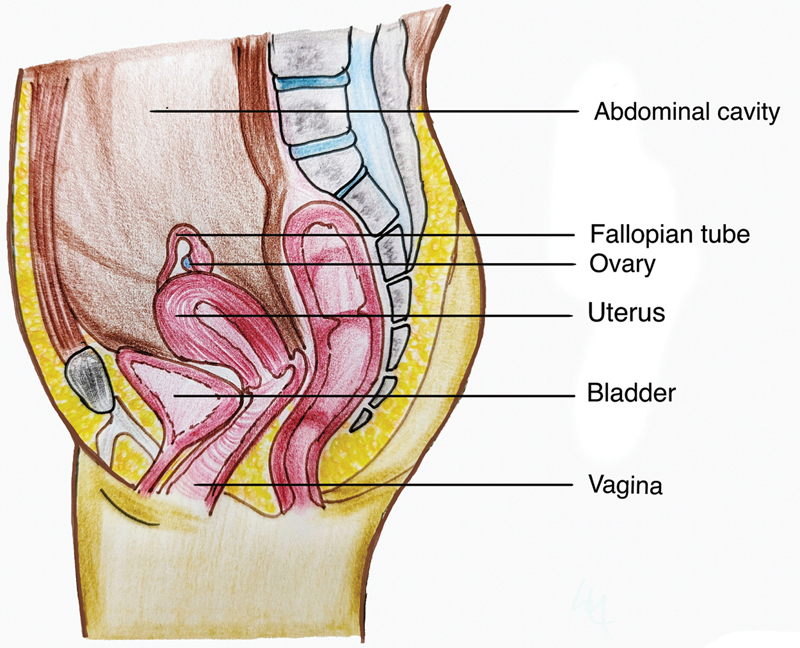
Sagittal section showing the location of normal vagina.

All procedures were performed in lithotomy position and with 4.0× magnification loupes. After catheterization, incisions were made to create a neovaginal canal ( Fig. 2 ) extending to the rectovesical space (approximately 6–10 cm). In the McIndoe vaginoplasty procedure, an intermediate-thickness skin graft harvested from the thigh was minimally meshed and secured over a vaginal mould with the dermal surface oriented toward the neovaginal cavity. The mould was then inserted into the surgically created space to facilitate graft adherence and maintain patency during the initial healing phase. The other procedures were done with or without the use of a preoperative Doppler marking of the pudendal perforators. The flaps were marked on either side based on the required length of the vaginal lining and subsequently elevated either superficially or deeply depending upon the procedures. The flaps were inverted and sutured to each other with the epidermis facing the lumen. Anchoring sutures were placed near the Pouch of Douglas, and the patency was maintained initially with a mould. The donor sites were closed primarily with or without drains. Sterile dressings were applied and changed regularly over the next 2 weeks. Patients were allowed to ambulate from day 3 post-op and were instructed on genital hygiene and regular mould use. The mould was changed every 2 weeks to 1 month by increasing the diameter to aid in further vaginal dilatation. Sexual intercourse was permitted 3 months following the procedure if the patency was adequately maintained with the mould by the patient. We have devised an Institutional Outcome Assessment Score, which evaluates four parameters: length of the vagina, diameter of introitus, quality of neovaginal skin, and pain during coitus ( Table 1 ), which was calculated by the end of 3 months postoperatively. Each parameter was scored across three categories:

**Table 1:** Institutional outcome assessment score

Parameters assessed	I	II	III
Length of vagina	Less than 5 cm	5–8 cm	7–10 cm
Diameter of introitus	Less than 2 cm	2–3 cm	3–5 cm
Quality of neovaginal skin	Uneven and contracted	Soft and uneven	Soft and supple
Pain during coitus	Frequent	Occasional	No pain

Note: Maximum score: 12; minimum score: 4. 10–12: excellent; 6–9: good; 4–6: fair; <4: poor.

- Vaginal length (<5 cm = 1, 5–8 cm = 2, 7–10 cm = 3).
- Introitus diameter (<2 cm = 1, 2–3 cm = 2, 3–5 cm = 3).
- Neovaginal skin quality (contracted = 1, soft/uneven = 2, soft/supple = 3).
- Pain during intercourse (frequent = 1, occasional = 2, none = 3).

The maximum score is 12, and the minimum of 4. A score of 10 to 12 is considered excellent, 6 to 9 is regarded as good, 4 to 6 as fair, and less than 4 is poor. We have established this scoring system that effectively assesses both the anatomical and functional characteristics of the vagina. By focusing on the attributes of an ideal neovagina, this system is a reliable and clinically valuable tool for evaluating postoperative outcomes in these patients.

### Case 1

**Fig. 3:**
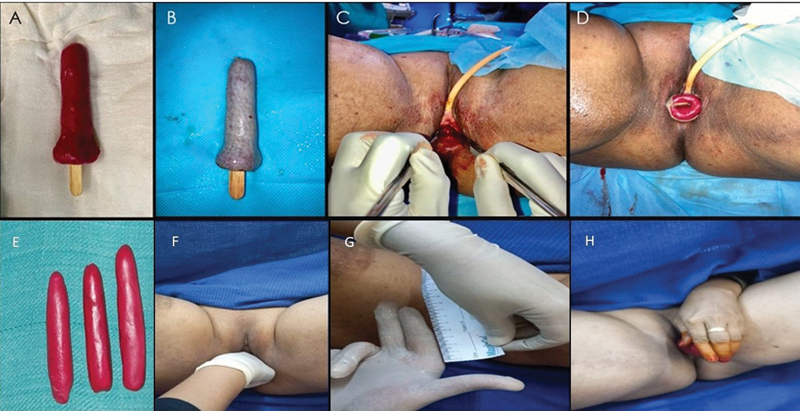
Intraoperative and postoperative images of McIndoe procedure. ( **A** ) Molded British dental compound. ( **B** ) Intermediate-thickness graft over mould. ( **C** ) Creation of neovagina. ( **D** ) Insertion of mould with graft for lining of neovagina. ( **E** ) Graded size of vaginal mould. ( **F, G** ) Per vaginal examination showing the length of neovagina, 6 months postoperatively. ( **H** ) Self-dilatation of vagina.

A 20-year-old unmarried woman presented with complaints of primary amenorrhea and absence of cyclical pain. Clinical examination revealed a vaginal dimple, with normal secondary sexual characteristics. Pelvic magnetic resonance imaging confirmed the presence of an aplastic uterus, cervix, and vagina, with normal ovaries. The patient was advised to undergo neovagina creation using the McIndoe procedure due to absent labia minora. The surgical procedure was performed under epidural and spinal anesthesia, in lithotomy position. Following urinary catheterization and anal packing, an incision was made over the area of the marked neovagina. Dissection was carried out using both blunt and sharp techniques within the rectovesical space until the Pouch of Douglas was reached, which served as the superior limit for dissection. Hemostasis was meticulously achieved throughout the process ( Fig. 3A–H ). An intermediate thickness skin graft, harvested from the patient's thigh, was tailored to exceed the dimensions of the preoperative mould. The graft was placed on sterile liquid paraffin-soaked gauze, with the dermal side oriented toward the raw surface, ensuring overlap of the graft margins. The mould supported by a sterile wooden stick, was subsequently inserted into the neovaginal cavity. Anal packing was removed, and a sterile dressing was applied. On postoperative day 5, the mould and urinary catheter were removed under anesthesia. Two weeks postoperatively, gradual dilatation was initiated. The patient was instructed to use the mould twice daily for the first 3 months and subsequently only at night for the next 3 months.

### Case 2

**Fig. 4:**
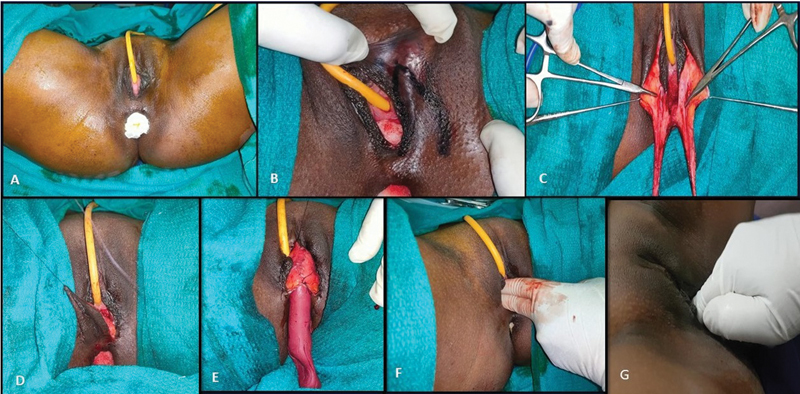
Intraoperative and postoperative images of labia minora two-flap vaginoplasty. ( **A** ) Preoperative photo of the perineum. ( **B** ) Labia minora flaps marked. ( **C, D** ) Flaps raised and tubed. ( **E** ) mould inserted. ( **F** ) Assessment of vaginal length following surgery. ( **G** ) Assessment of vaginal length, 6 months postoperatively.

**Fig. 5:**
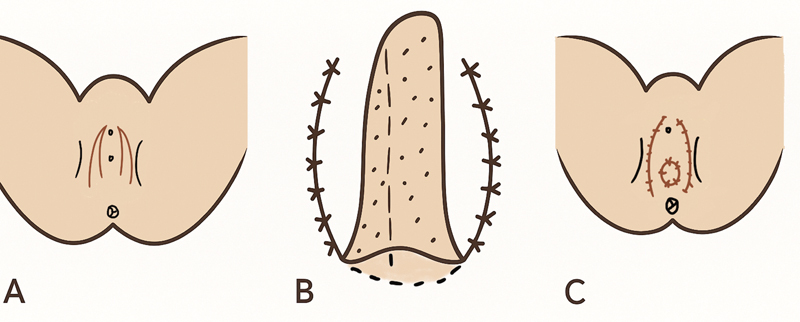
Diagrammatic representation of labia minora two-flap vaginoplasty. ( **A** ) Flap marking. ( **B** ) Tubing of flaps and creation of neovaginal lining. ( **C** ) Closure of donor sites.

A 24-year-old female was clinically and radiologically diagnosed with MRKH syndrome. She underwent vaginoplasty with the labia minora two-flap technique ( Fig. 4A–F ). Flaps of dimension 5 × 3 cm were marked over the bilateral labia minora and preputial skin with methylene blue. The labia minora flaps were elevated along with the preputial skin as a single unit on each side, inverted, and sutured together with 3–0 polyglactin to create the neovaginal lining ( Fig. 5A–C ). An anchoring stitch was placed adjacent to the Pouch of Douglas, and donor sites were closed primarily with 3–0 poliglecaprone. A mould was inserted postoperatively, with the first evaluation conducted on day 2, followed by regular dressing changes. Gradual neovaginal dilatation commenced 2 weeks post-surgery, with continuous mould use for 3 months and progressive adjustments of the dilators every 2 weeks to 1 month.

### Case 3

**Fig. 6:**
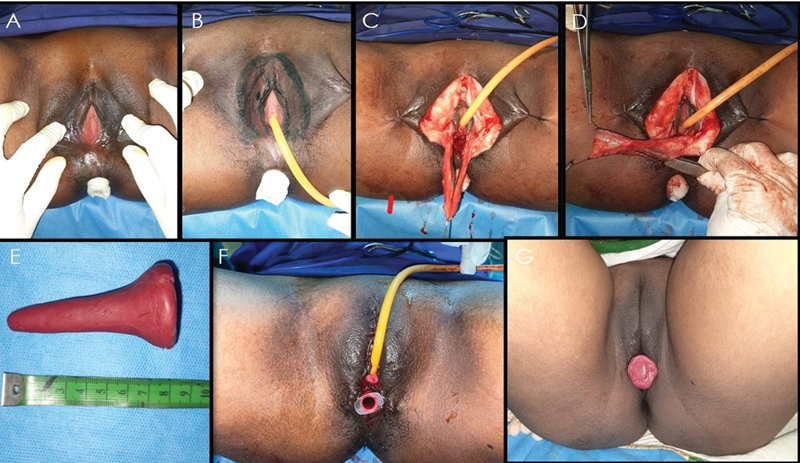
Intraoperative and postoperative images of horseshoe modification of labia minora flap. ( **A** ) Preoperative photo of perineum showing vaginal agenesis. ( **B** ) Marking of horseshoe flap. ( **C, D** ) Horseshoe flap raised and tubed. ( **E** ) mould made according to the length of neovagina. ( **F** ) Donor site closed primarily and mould inserted into neovagina. ( **G** ) One-month postoperative picture.

**Fig. 7:**
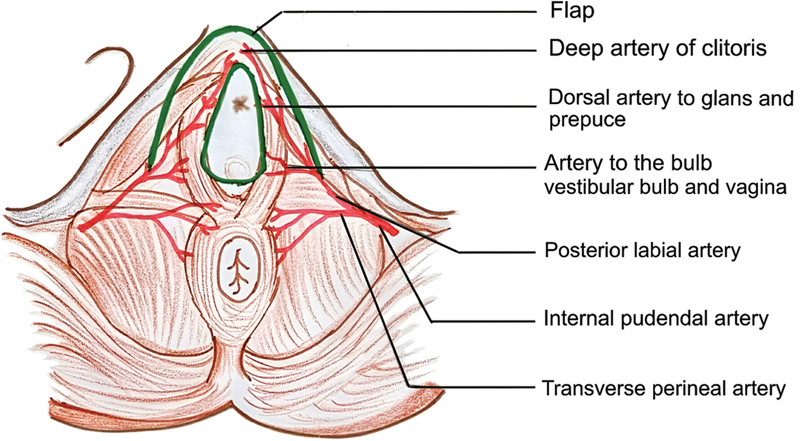
Blood supply of horseshoe modification of labia minora flap.

**Fig. 8:**
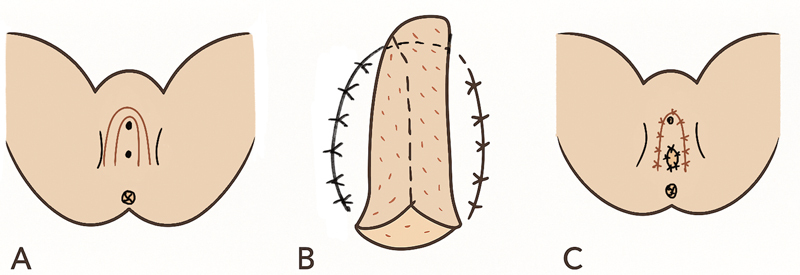
Diagrammatic representation of horseshoe modification of labia minora flap. ( **A** ) Flap marking. ( **B** ) Tubing of the flap in the neovagina. ( **C** ) Closure of donor site.

A 21-year-old female with MRKH syndrome underwent vaginoplasty with a horseshoe labia minora flap ( Fig. 6A–G ). In the lithotomy position, following urinary catheterization and anal packing, a posteriorly based horseshoe flap was marked ( Fig. 7 ), raised, inverted, and sutured at both ends, then anchored to the neovaginal base. Donor sites were closed with 3–0 poliglecaprone. A schematic diagram illustrating the horseshoe flap, including the preoperative marking and donor-site closure, is depicted in Fig. 8 (A–C) . The mould was inserted into the neovagina, and the same postoperative protocol as previously described was followed.

### Case 4

**Fig. 9:**
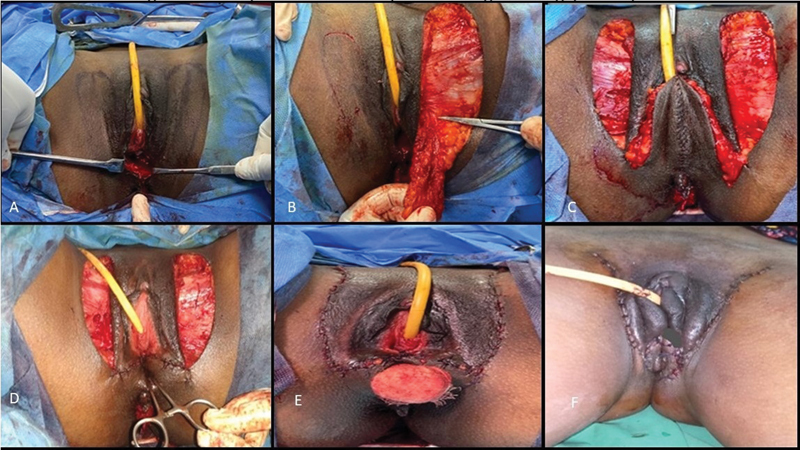
Intraoperative and postoperative images of Singapore flap. ( **A** ) Marking of Singapore flap. ( **B, C** ) Raising of flap on either side and suturing it together. ( **D** ) Tubing in the neovagina. ( **E** ) Closure of donor site. ( **F** ) Immediate postoperative image.

A 23-year-old female with primary amenorrhea was diagnosed with MRKH syndrome and underwent vaginoplasty using the Singapore flap technique. The flap is vascularized by branches of the internal pudendal artery and innervated by branches of the pudendal nerve. Sensate skin flaps (8 × 3 cm) were marked bilaterally on the inner thigh, raised, tunneled toward the neovagina, sutured together, and anchored to their base. Donor sites were closed with 3–0 poliglecaprone, and a mould was inserted. The postoperative protocol followed standard guidelines ( Fig. 9A–F ).

### Case 5

**Fig. 10:**
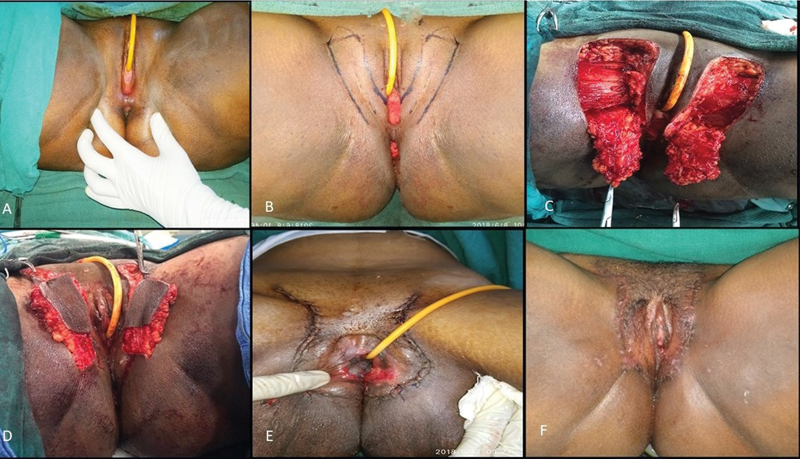
Intraoperative and postoperative images of islanded pudendal flap. ( **A** ) Preoperative photo of perineum showing vaginal agenesis. ( **B** ) Marking of islanded pudendal flap. ( **C, D** ) Flap elevation. ( **E** ) Suturing of both flaps and tubing in the neovagina. ( **F** ) Late postoperative image.

A 27-year-old female with primary amenorrhea and exhibiting normal secondary sexual characteristics was diagnosed with MRKH syndrome and underwent vaginoplasty using an islanded pudendal thigh flap ( Fig. 10A–F ). The surgical technique followed the previously described approach, with the primary distinction being the islanding of the flap to enhance the vascularity and mobility of the flap.

## Results

This study included 20 patients diagnosed with MRKH syndrome presenting with vaginal agenesis, all of them were operated by the same surgical team ( Table 2 ). Seven patients underwent McIndoe procedure, two using the Singapore flap technique, one underwent the islanded pudendal flap technique, two underwent the labia minora flap technique, and eight underwent the horseshoe modification of the labia minora flap. Patient ages ranged from 18 to 28 years, and all were unmarried at the time of surgery. Ten patients were married within 6 months postoperatively.

**Table 2:** Demographic data

S. No	Age (y)	Procedure	Follow-up period (mo)	Institutional outcome assessment score	Complications
1	21	Horseshoe flap	12	12	Nil
2	18	Labia minora flap	16	11	Nil
3	20	McIndoe procedure	12	9	Partial graft loss
4	28	Horseshoe flap	18	12	Nil
5	24	Labia minora flap	18	11	Nil
6	22	McIndoe procedure	12	8	Minimal pain
7	23	Singapore flap	12	9	Nil
8	25	Horseshoe flap	12	12	Nil
9	19	Singapore flap	12	11	Nil
10	17	Horseshoe flap	12	12	Nil
11	20	McIndoe procedure	14	10	Graft contracture
12	21	Horseshoe flap	12	11	Nil
13	26	McIndoe procedure	16	10	Nil
14	27	Islanded pudendal flap	14	10	Minimal pain
15	19	McIndoe procedure	12	9	Nil
16	18	Horseshoe flap	18	11	Nil
17	25	Horseshoe flap	16	12	Nil
18	22	McIndoe procedure	12	8	Nil
19	27	McIndoe procedure	12	9	Nil
20	26	Horseshoe flap	14	12	Nil

Following McIndoe procedure, one patient (14.28%) experienced partial graft loss, successfully managed through conservative measures, while another (14.28%) developed graft contracture, which required surgical release and regrafting. No other significant complications were observed in the remaining patients. Postoperatively, all patients used a mould for 3 months. The vulva appeared normal with a sensate introitus and fair lubrication, likely due to sebaceous secretions. Some patients reported aesthetic concerns due to the absence of labia minora folds.

**Fig. 11:**
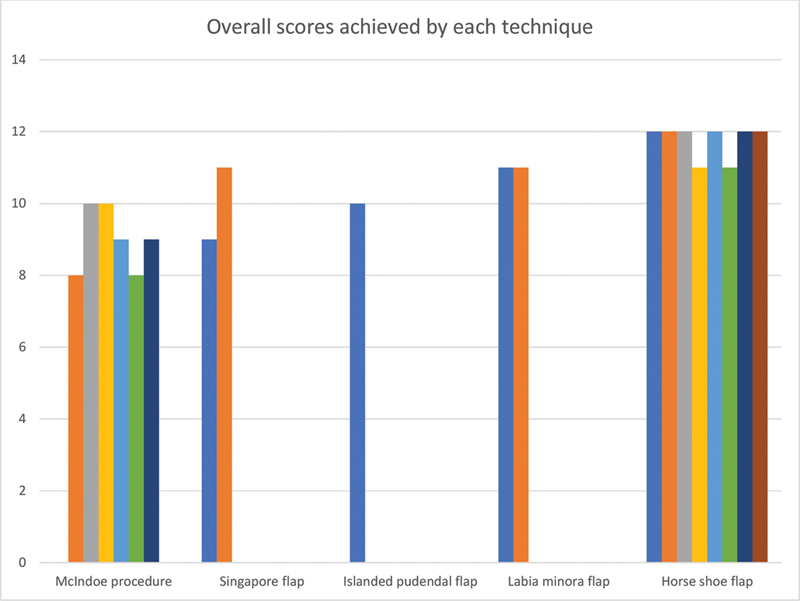
Graph depicting overall score achieved by each technique.

The mean follow-up period was 18 months. The postoperative vaginal length ranged from 6 to 10 cm, with an average of 8 cm. Institutional outcome assessment scores were determined based on predefined parameters ( Fig. 11 ) and calculated at 3 months postoperatively.

## Discussion

Vaginoplasty presents a significant surgical challenge due to its structural and functional complexities. Currently, no single gold-standard technique exists for managing MRKH syndrome, as every approach has its own challenges and limitations. The first nonsurgical method, Frank's technique, involves continuous mould application against a vaginal dimple or pouch to facilitate neovaginal formation. 11

The five most frequently performed vaginoplasty techniques include: (1) McIndoe procedure, (2) pudendal thigh flap (modified Singapore flap), (3) islanded pudendal thigh flap, (4) labia minora two-flap technique, and (5) horseshoe modification of the labia minora flap. Each technique carries distinct advantages and drawbacks. Although McIndoe procedure is simple and effective, complications such as graft loss, contracture, vaginal canal narrowing, unstable scarring, dryness, and the requirement for regular dilatation limit its widespread use. 12 13

The pudendal thigh flap, also known as the Singapore flap, first described by Wee, offers a highly vascularized and pliable tissue that conforms well to the vaginal cavity. However, they are susceptible to apical necrosis, aside from creating a noticeable scar in the groin, altering the esthesis of the external genital. Additionally, bulky flaps contribute to complications such as delayed wound healing and dehiscence. 8 14 Myocutaneous flaps like gracilis or rectus abdominis are excessively bulky and necessitate extensive dissection.

Intestinal transfers, including rectosigmoid or sigmoid colon vaginoplasty (free or pedicled), provide excellent depth and natural lubrication but pose significant morbidity risks. These include additional abdominal surgery, intestinal anastomosis, visible scarring, lack of vaginal sensation, and persistent mucous discharge, leading to patient discomfort. There is also a rare risk of malignant transformation. 15 16

Comparative analysis in this study suggests that the horseshoe modification of the labia minora flap is a superior alternative for vaginal reconstruction in primary vaginal atresia associated with MRKH syndrome. The Singapore and lotus petal flaps are bulky and often necessitate secondary thinning procedures, whereas the labia minora flap is thin and pliable, eliminating the need for additional surgical interventions. Similar approaches using bilateral labia minora flaps have been reported by Flack et al 17 and the horseshoe flap by Purushothaman. 18

The labia minora provides an optimal neovaginal lining due to its histological similarity, lubrication, and sensory function at the introitus. Initially, the postoperative neovaginal length measured 6 cm, increasing to 8 cm following regular dilatation. The labia minora flap offers several advantages, including soft, supple skin, a robust axial pattern, concealed donor scars, minimal contracture risk, and technical simplicity. Its strong vascular pedicle minimizes ischemia and flap necrosis.

Minor limitations include the restricted availability of donor skin, the necessity for consistent dilatation, and the potential for aesthetic concerns due to the absence of labia minora folds. Nevertheless, this technique remains a simple, reliable, and effective option with minimal morbidity.

## Conclusion

Based on the outcomes observed in our series, the horseshoe modification of the labia minora flap provides an optimal and esthetically refined approach to vaginal reconstruction in MRKH syndrome, ensuring a sensate neovagina. Its thin and pliable nature obviates the need for secondary procedures while facilitating adequate vaginal length with minimal contracture risk and low donor-site morbidity. This technique adheres to the fundamental principles of plastic surgery by achieving both functional and structural restoration. Moreover, its technical simplicity and relatively short learning curve render it a viable option for both experienced and novice plastic surgeons.
